# Unstaged cancer in the United States: a population-based study

**DOI:** 10.1186/1471-2407-11-402

**Published:** 2011-09-21

**Authors:** Ray M Merrill, Arielle Sloan, Allison E Anderson, Karem Ryker

**Affiliations:** 1 Department of Health Science at Brigham Young University, Provo, Utah, USA

**Keywords:** cancer, incidence, population-based, relative survival, SEER, unstaged

## Abstract

**Background:**

The current study examines unstaged disease for 18 cancer sites in the United States according to the influence of age, sex, race, marital status, incidence, and lethality.

**Methods:**

Analyses are based on 1,040,381 male and 1,011,355 female incident cancer cases diagnosed during 2000 through 2007. Data were collected by population-based cancer registries in the National Cancer Institute's Surveillance, Epidemiology, and End Results Program.

**Results:**

The level of unstaged disease was greater in more lethal cancers (e.g., liver, esophagus, and pancreas) compared with less deadly cancers (i.e., colon, urinary bladder, and female breast). Unstaged disease increased with age and is greater among non-married patients. Blacks compared with whites experienced significantly higher levels of unstaged cancers of the stomach, rectum, colon, skin (melanoma), urinary bladder, thyroid, breast, corpus, cervix, and ovaries, but lower levels of unstaged liver, lung and bronchial cancers. Males compared with females experienced significantly lower levels of unstaged cancers of the liver, pancreas, esophagus, and stomach, but significantly higher levels of unstaged lung and bronchial cancer and thyroid cancer. The percent of unstaged cancer significantly decreased over the study period for 15 of the 18 cancer sites.

**Conclusion:**

Tumor staging directly affects treatment options and survival, so it is recommended that further research focus on why a decrease in unstaged disease did not occur for all of the cancer sites considered from 2000 to 2007, and why there are differential levels of staging between whites and blacks, males and females for several of the cancer sites.

## Background

Cancer staging, an important prognostic indicator, provides direction on an appropriate course of treatment. However, each year a small percentage of newly diagnosed cancers are not assigned a tumor stage. In a study involving postmenopausal women with breast cancer in the United States (U.S.), increased age was significantly associated with decreased staging of the tumor and with fewer auxiliary lymph node dissections in women aged 70 years and older [1]. The association between older age and decreased staging of the tumor was also identified in studies involving prostate cancer, as well as colon and rectal cancers in the U.S. [2-5]. In addition to the association between advancing age and unknown tumor stage, studies revealed that more vulnerable patients, such as minority groups, patients requiring more complex care needs in long-term care settings, those with lower education levels, and the uninsured are more likely to have unstaged cancer [2-6].

Components of staging include tumor size (T), number of lymph nodes (N), and metastases (M). When examining the contribution that missing TNM information has on unstaged colon and rectal cancers, Worthington and colleagues observed that M was missing for most colon and rectal cancer cases with unstaged disease [4]. Refusal to undergo diagnostic testing and treatment or an inoperable tumor possibly contributed to missing information. The authors also suggested that unknown stage may have resulted if patients were only treated with endoscopic therapy and local excision, or cared for in areas where definite staging was not available.

Thus far, the study of unstaged disease has focused primarily on cancers of the breast, prostate, colon and rectum. The purpose of this study is to identify the level of unknown-staged disease for 18 major cancer sites in the U.S., identify changes in the level of unknown staging for the selected cancer sites from 2000 through 2007, identify the influence of age, sex, race, and marital status on the level of unstaged disease for the selected cancer sites, and identify how the incidence rate and lethality of the cancer is associated with the level of patient staging for that disease.

## Methods

### Data

Analyses are based on 2,726,147 newly diagnosed cancer cases during 2000 through 2007 collected from medical records at hospitals and other facilities by 17 population-based cancer registries in the Surveillance, Epidemiology, and End Results (SEER) Program of the National Cancer Institute [7]. The SEER Program was established in response to the National Cancer Act of 1971, which mandated public health surveillance of cancer in the U.S. for use in prevention, diagnosis, and treatment of cancer. The SEER Program began collecting data on cancer cases on January 1, 1973, with seven registries (Connecticut, Iowa, New Mexico, Utah, and Hawaii, and the metropolitan areas of Detroit and San Francisco-Oakland). In the following two years the metropolitan area of Atlanta and the 13-county Seattle-Puget Sound area were added. In 1992, the SEER Program was expanded to increase coverage of minority populations to include 10 primarily black rural counties in Georgia, the Alaska Native population, Los Angeles County, and four counties in the San Jose-Monterey area south of San Francisco. In 2000, the SEER Program further expanded coverage to include Kentucky and the remaining counties in California (Greater California); in addition, New Jersey and Louisiana once again became participants (Surveillance, Epidemiology, and End Results [SEER] Program [http://www.seer.cancer.gov]) [8]. These areas cover 26% of the U.S. population (23% of African Americans, 40% of Hispanics, 42% of American Indians and Alaska Natives, and 59% of the Asian/Pacific Islander population) [8].

Among the newly diagnosed cancer cases, 2,493,516 (91%) were classified as non-Spanish-Hispanic-Latino (83% white, 10% black, 1% American Indian/Alaska Native, and 6% Asian or Pacific Islander) and 232,631 (9%) were classified as Spanish-Hispanic-Latino (97% white, 1% black, 1% American Indian/Alaska Native, and 1% Asian or Pacific Islander).

The tumor registries participating in the SEER Program routinely abstract records of all cancer patients in hospitals, clinics, nursing homes, and other health service units that provide diagnostic or treatment services; from private pathology laboratories and radiotherapy units; and from death certificates. Data collected by the tumor registries include patient demographics, tumor characteristics, morphology, diagnostic information, extent of disease, first course of treatment, and active patient follow-up of vital status including cause of death. Cancers are coded according to the International Classification of Disease for Oncology Second Edition (ICD-O-2) [9]. This study uses the SEER historic summary stage classification to identify unstaged disease.

Cancer frequencies, rates, and survival probabilities were calculated using the SEER Survival System (SEER*Stat) (Surveillance Research Program, National Cancer Institute SEER*Stat software [http://www.seer.cancer.gov/seerstat] version 6.6.2).

### Variables

Eighteen cancer sites were selected for study: liver, pancreas, esophagus, stomach, lung and bronchus, soft tissues, prostate, kidney and renal pelvis, oral cavity and pharynx, colon, rectum, melanoma of the skin, urinary bladder, thyroid, cervix uteri, breast (female), corpus uteri, and ovary. These sites were selected because they provide a representation of more and less common cancers, more and less lethal cancers, and cancers specific to the male and female genital systems. In addition, age (0-59, 60-79, and 80 years or older), sex, race (whites, blacks), and marital status (married [or cohabitating] or single), ethnicity, and Appalachia residence status were included as variables in the study.

### Statistical Techniques

Cancer site-specific percentages of unstaged disease were calculated by dividing the age-adjusted malignant incidence rate of unstaged disease by the total age-adjusted incidence rate. Rates were age-adjusted using the 2000 U.S. standard population. In a multiple regression model, the percentage of unstaged cancer was regressed on calendar year (2000 through 2007), race (white, black), and sex, and evaluated for statistical significance using the F test. The percentage of unstaged cancer was also regressed on age, sex, race, and marital status separately for each of the selected cancer sites. Interaction terms were assessed in the model between age, sex, race, and marital status with cancer site. Multiple regression models were used to evaluate whether the level of unstaged cancer was associated with malignant incidence rates for the selected cancers and also if the level of unstaged cancer was associated with the cancer site-specific five-year relative survival, adjusting for age, sex, and race.

We used relative survival to circumvent the problem associated with tumor registries of inaccurate or unavailable death certificates and the uncertainty about the cause of death [10-12]. Relative survival is the ratio of the proportion of observed survivors in a cohort of cancer patients to the proportion of expected survivors in a comparable set of cancer free individuals. The formulation is based on the assumption of independent competing causes of death. The relative survival adjusts for the general survival of the U.S. population for the race, sex, age, and date at which the age was coded. If age, race, sex, or year information is missing, that individual is excluded from the analysis. Standard case selection criteria employed by the SEER Program were used; that is, cases were selected if they were actively followed and had malignant behavior and a known age. Cases were excluded if they were a second or later primary. Death certificate-only and autopsy-only cases were also excluded. Patients diagnosed between 2000 and 2007 were included, with follow-up through 2007.

Analyses were performed using the Statistical Analysis System (SAS) software, version 9.2 (SAS Institute Inc., Cary, NC, USA, 2007).

## Results

**Figure 1:**
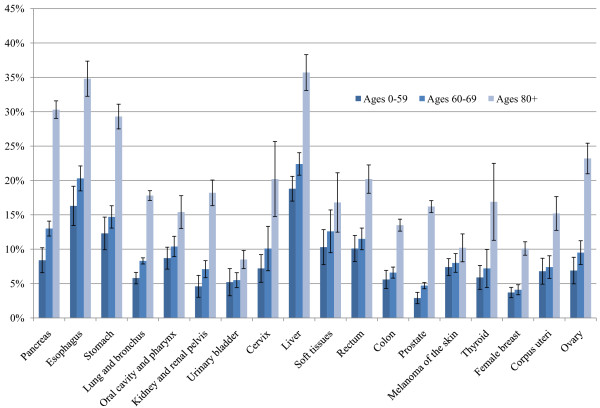
**Percentages of Unstaged Disease for Selected Cancer Sites According to Age**. Adjusted for sex. Source: Surveillance, Epidemiology, and End Results (SEER) 2000-2007.

**Figure 2:**
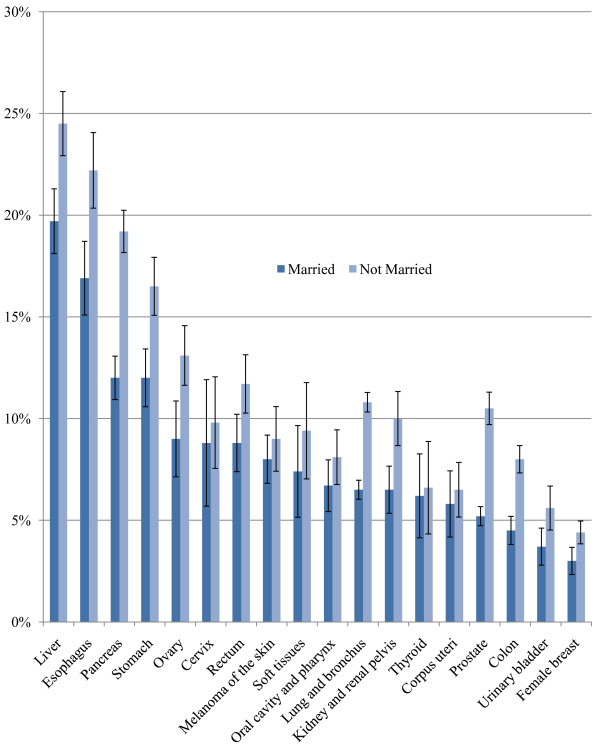
**Percentages of Unstaged Disease for Selected Cancer Sites According to Marital Status**. Adjusted for age and sex. Source: Surveillance, Epidemiology, and End Results (SEER) 2000-2007.

**Figure 3:**
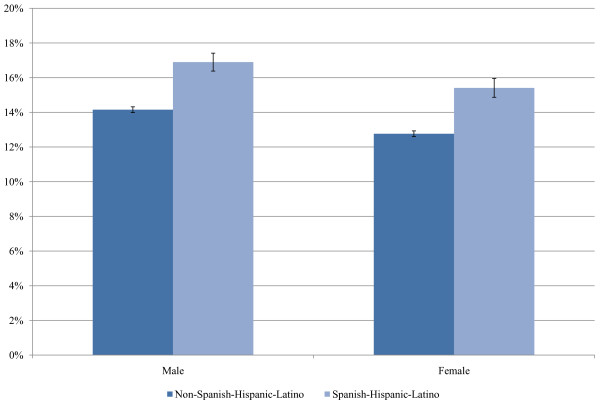
**Percentages of Unstaged Disease According to Ethnicity and Sex**. Adjusted for age. Source: Surveillance, Epidemiology, and End Results (SEER) 2000-2007.

**Figure 4:**
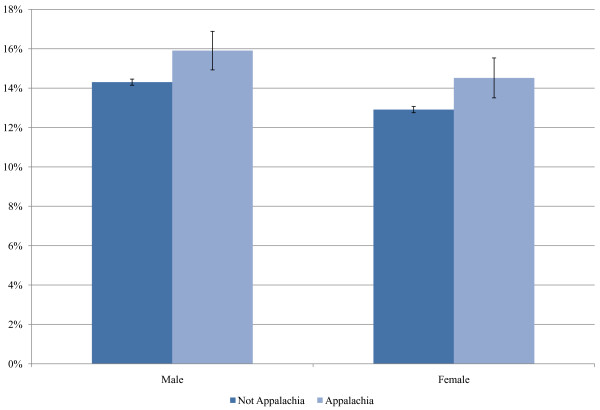
**Percentages of Unstaged Disease According to Appalachia Residence and Sex**. Adjusted for age. Source: Surveillance, Epidemiology, and End Results (SEER) 2000-2007.

The level of unstaged disease is presented for 18 selected cancer sites for white and black patients in Figure 1. The highest levels of unstaged disease occur in cancers of the liver, esophagus, and pancreas and the lowest levels involve cancers of the colon, urinary bladder, and female breast. Unstaged disease for white and black patients increases sharply with age, with the greatest percent increase observed in cancers of the pancreas, lung and bronchus, kidney and renal pelvis, prostate, and ovaries. For several of the cancer sites, there is also a significant positive association between staging and being married (Figure 2). In addition, for all racial groups, patients of Spanish-Hispanic-Latino descent (Figure 3) and residents of Appalachia (Figure 4) were significantly more likely to have their cancer unstaged.

Trends in unstaged disease for white and black patients are presented for each of the selected cancer sites in Table 1. A significant decrease in unstaged disease occurred for each of the cancers, with the exception of malignancies of the oral cavity and pharynx, melanoma of the skin, cervix, and the thyroid, where no significant change occurred, and stomach cancer, where an increase in unstaged disease occurred. Blacks had significantly higher levels of unstaged cancers of the stomach, rectum, colon, skin (melanoma), urinary bladder, kidney and renal pelvis, thyroid, breast, corpus, cervix, and ovaries. On the other hand, blacks with liver cancer or lung and bronchial cancer were significantly more likely to have their tumor staged. Males compared with females experienced significantly lower levels of unstaged cancers of the liver, pancreas, esophagus, and stomach, but significantly higher levels of unstaged lung and bronchial cancer and thyroid cancer. There was a significant statistical interaction involving sex and race in the model for thyroid cancer. Among males, blacks had a significantly higher level of unstaged thyroid cancer (2.04%, P = 0.007), but among females, there was no significant difference in the level of unstaged thyroid cancer.

**Table 1:** Estimated effects of calendar year, race, and sex on percent of selected site-specific unstage cancer

	Estimate*	P Value^†^
Liver		
Year	-1.28%	< 0.001
Race (Black vs. White)	-2.67%	< 0.001
Sex (Male vs. Female)	-1.77%	0.012

Pancreas		
Year	-0.77%	< 0.001
Race (Black vs. White)	-0.24%	0.675
Sex (Male vs. Female)	-1.65%	0.006

Esophagus		
Year	-0.66%	0.003
Race (Black vs. White)	1.92%	0.051
Sex (Male vs. Female)	-4.84%	< 0.001

Stomach		
Year	0.32%	0.008
Race (Black vs. White)	2.07%	< 0.001
Sex (Male vs. Female)	-2.11%	< 0.001

Lung and bronchus		
Year	-0.32%	< 0.001
Race (Black vs. White)	-0.72%	0.006
Sex (Male vs. Female)	0.51%	0.047

Soft tissues		
Year	-0.36%	0.026
Race (Black vs. White)	-0.51%	0.470
Sex (Male vs. Female)	0.41%	0.561

Rectum		
Year	-0.29%	0.019
Race (Black vs. White)	4.48%	< 0.001
Sex (Male vs. Female)	-0.32%	0.552

Oral cavity and pharynx		
Year	0.12%	0.462
Race (Black vs. White)	-0.06%	0.930
Sex (Male vs. Female)	-0.20%	0.780

Colon		
Year	-0.18%	< 0.001
Race (Black vs. White)	1.58%	< 0.001
Sex (Male vs. Female)	0.08%	0.734

Kidney and renal pelvis		
Year	-0.36%	< 0.001
Race (Black vs. White)	1.19%	< 0.001
Sex (Male vs. Female)	0.19%	0.524

Prostate		
Year	-0.49%	< 0.001
Race (Black vs. White)	0.52%	0.108

Melanoma of the skin		
Year	0.14%	0.666
Race (Black vs. White)	4.63%	0.004
Sex (Male vs. Female)	-1.89%	0.206

Urinary bladder		
Year	-0.19%	0.030
Race (Black vs. White)	2.29%	< 0.001
Sex (Male vs. Female)	-0.49%	0.200

Thyroid		
Year	-0.13%	0.155
Race (Black vs. White)	1.13%	0.010
Sex (Male vs. Female)	1.02%	0.019

Female Breast		
Year	-0.18%	< 0.001
Race (Black vs. White)	0.89%	< 0.001

Corpus Uteri		
Year	-0.26%	0.002
Race (Black vs. White)	2.62%	< 0.001

Cervix		
Year	-0.16%	0.295
Race (Black vs. White)	2.64%	0.002

Ovary		
Year	-0.39%	0.019
Race (Black vs. White)	3.69%	< 0.001

Data source: Surveillance, Epidemiology and End Results (SEER) Program, 2000-2007.

*Estimates in each model are adjusted for calendar year, race, and sex.

^†^Based on the F value.

There was no significant association between the level of unstaged disease and incidence rates for the selected cancer sites, with the exception of white females (Table 2). For this group, cancer sites with higher incidence rates had significantly lower unstaged disease. We also assessed the association between the level of unstaged disease and five-year relative survival for the selected cancer sites. For males and females, whites and blacks, cancer sites with greater five-year survival had lower unstaged disease.

**Table 2:** Site-specific percentage of unstage cancer, malignant cancer incidence rates, and 5-year relative survival according to race and sex

	White Males			White Females			Black Males			Black Females		
	**Unstage** %*	**Malignant** **Incidence** **Rate^†^**	**5-yr Survival** **%^‡^**	**Unstage** %*	**Malignant** **Incidence** **Rate^†^**	**5-yr Survival** **%^‡^**	**Unstage** %*	**Malignant** **Incidence** **Rate^†^**	**5-yr Survival** **%^‡^**	**Unstage** %*	**Malignant** **Incidence** **Rate^†^**	**5-yr Survival** **%^‡^**

Liver	20.3	7.9	10.9	24.0	2.5	12.9	17.8	12.8	6.4	18.9	3.6	9.9

Pancreas	14.4	13.1	4.4	15.7	10.2	4.6	13.9	16.5	3.9	16.1	14.3	5.2

Esophagus	13.9	8	14.1	21.1	1.9	14.2	16.7	9.6	7.2	22.6	3.0	10.3

Stomach	12.1	9.9	18.6	14.9	4.7	23.5	14.5	17.2	19.5	15.6	9.0	25.4

Lung and bronchus	8.6	78.9	12.9	8.4	54.9	17.7	8.2	106.1	10.7	7.4	54.3	14.4

Soft tissues	7.7	3.9	66.5	7.4	2.7	66.5	8.6	3.6	60.2	6.7	3.1	63.4

Rectum	7.1	12.7	60.4	7.9	7.6	61.6	11.3	12.4	51.9	11.8	8.5	57.5

Oral cavity and pharynx	6.3	15.8	58.2	6.6	6.1	61.3	6.5	16.9	33.1	6.9	5.7	49.9

Colon	5.2	40.5	61.6	5.3	32	61.3	7.0	52.7	53.3	6.8	42.3	53.8

Kidney and renal pelvis	5.2	19.1	63.9	5.2	9.7	64.1	7.0	21.3	60.8	6.7	10.4	64.2

Prostate	4.9	158	93.4				5.4	248.2	88.9			

Melanoma of the skin	3.8	28.9	86.8	3.8	18.6	92.2	8.3	1.1	65.1	11.1	0.9	76.1

Urinary bladder	3.2	40.6	80.9	4.0	9.9	75.8	5.4	20.5	67.7	5.2	7.6	53.4

Thyroid	2.0	5.1	93.5	2.1	14.7	97.4	3.7	2.7	88.8	2.4	8.2	94.9

Female Breast				2.1	130.7	86.1				3.0	119.1	73.5

Corpus Uteri				3.3	24	86				5.7	19.2	60.8

Cervix				4.8	8.3	71.3				7.4	10.7	61.1

Ovary				7.1	14.1	42.9				10.8	10.1	38.8

Spearman's Rho		-0.38	-0.92		-0.62	-0.93		-0.31	-0.80		-0.32	-0.70

P Value		0.179	< 0.001		0.008	< 0.001		0.283	< 0.001		0.216	0.002

Data source: Surveillance, Epidemiology and End Results (SEER) Program, 2000-2007.

*Age-adjusted to the 2000 US standard population.

^†^Age-adjusted to the 2000 US standard population and expressed per 100,000.

^‡^Relative survival, which is a net survival measure representing cancer survival in the absence of other causes of death.

## Discussion

Percentages of unstaged disease increased significantly with age for each of the 18 selected cancer sites, as consistent with previous studies [1-5,13]. Age and comorbid conditions limit one's ability to undergo testing and examinations. Neither elderly patients nor their family members may consent to a diagnostic workup [3]. This is especially the case when older patients have competing health problems that limit disease investigation. In a study involving elderly postmenopausal breast cancer patients, the authors concluded that compromised health status associated with age may have precluded patients from obtaining certain prognostic information (e.g., Axillary lymph node dissection [AxLND] for breast cancer patients), which in turn can limit treatment options [1].

Married individuals were significantly more likely to receive a cancer staging, after adjusting for age, sex, and race. Previous studies have not specifically considered the influence of marital status on unknown stage. However, this result is consistent with findings from studies showing that married cancer patients tend to be identified at an earlier stage of disease, experience fewer comorbid conditions, and have better prognosis [14-16]. One study reported that married individuals have higher socioeconomic status and social support, which in turn leads to higher survival than non-married individuals [17]. Older women who are assigned an advanced tumor stage or no tumor stage at diagnosis are more likely to be widowed than younger women, who will also have a higher chance of survival. Another study found that women who were married, white or of higher socioeconomic status were more likely to undergo mammography and receive pap testing than single women [16]. Married women enjoy the benefit of a combined income and a stable partner, which increases their likelihood of being able to afford appropriate medical services.

The higher percentage of unstaged cases who are of Spanish-Hispanic-Latino descent or who reside in Appalachia may be explained, at least in part, by lower levels of health insurance, which limits one's ability to undergo testing and examinations. Culture may also influence the patient's willingness to consent to a diagnostic workup. In addition, a thorough cancer workup is more limited among patients of poorer overall health status. Further research focusing on cancer staging by ethnicity and Appalachia status is warranted.

The percentage of cases receiving an unknown stage assignment decreased over the study period for 15 of the 18 cancer sites considered. Other studies focusing on colon and rectal cancers and prostate cancer also observed a decrease in unknown staging [2,4]. Reasons for this increase in staging may include physician education, introduction of less invasive staging procedures, and adoption of a collaborative staging system by SEER in 2004, which combines and standardizes information using computer algorithms to assign a stage.

This study also revealed a higher percentage of unstaged rectal cancer than colon cancer. The authors of another study likewise observed a higher percentage of unstaged rectal cancer than colon cancer, attributing this to the limited availability of endoscopic ultrasounds often required for staging rectal cancer [2]. However, the same screening methods are typically used to detect both rectal and colon cancer, and some of these methods (e.g., sigmoidoscopy) are more likely to detect cancers of the rectum or distal colon.

Two previous SEER data-based studies identified that the percentage of unstaged colorectal cancer was significantly lower for males than females [3,4]. In the current study, we found no significant difference in the percentage of unstaged colon or rectal cancers between males and females, after adjusting for age and race. However, in rerunning the models without adjusting for these variables, a similar result was found to those in the previous studies. The large difference in life expectancy between males and females in the U.S. emphasizes the need to adjust for the potential confounding effect of age. In addition, some researchers have identified that older males in the U.S. tend to have higher socioeconomic status than their female counterparts, and that older women are more likely to be insured by Medicaid [3]. Patients with advanced age and covered by Medicaid have a higher probability of having their cancer recorded as unknown.

Lower screening rates among females for esophageal and stomach cancers may explain significantly higher rates of unstaged disease for females compared to males after adjusting for age and race. For example, one study involving stomach cancer reported that 32.5% of men compared with 23.5% of women underwent screening for the disease [18]. Of those who were screened, approximately half were screened at work, which suggests that unemployed women are less likely to be screened. (Note that in the U.S., only high risk individuals are recommended to pursue esophageal or stomach cancer screening [19].) Also, because stomach cancer is more prevalent among males than females, females are less motivated to undergo screening for this disease--especially since no routine stomach cancer screening method exists [19].

Black males were less likely than white males to be assigned a tumor stage for cancers involving the stomach, rectum, colon, kidney and renal pelvis, and thyroid. Black females were less likely than white females to be assigned a tumor stage for cancers involving the breast, corpus, cervix, and ovaries. Higher levels of unstaged disease among blacks have been observed in other studies [4,6,20]. A higher level of unstaged disease among blacks compared with whites is consistent with their tendency to be diagnosed at a later stage, to have more comorbid conditions, and to experience poorer survival rates [20,21]. Poorer survival among unstaged patients may be explained by lower levels of treatment [1,4-6,12,22] In addition, unstaged patients who do not receive surgery tend to experience more severe comorbidities than patients that receive treatment [1]. Although unstaged disease influences treatment outcomes, age and comorbid conditions are contributing factors since they are negatively associated with cancer-directed therapy [5].

One study found that uninsured patients are less likely to receive a tumor stage than insured patients [6]. Another study observed that poorer socioeconomic status among blacks is associated with generally more advanced cancers of the colon or rectum, lung, and cervix [23], which in turn could explain higher levels of unstaged disease [5]. Lower socioeconomic status is likely associated with blacks seeking medical care later in the disease process, which in turn influences their higher levels of unstaged disease. With a greater proportion of unstaged disease among minorities, race-stage information may be biased.

Only for white females were the cancer sites with higher incidence rates significantly associated with lower unstaged disease. This may be because of increased cost associated with rare cancer diagnosis and treatment [24]. Individuals with rare cancers will probably need more sophisticated diagnostic procedures in general, and those who refuse such procedures because of age or monetary factors will be more likely to receive an unknown tumor stage diagnosis.

We observed a strong and consistent association between cancer sites with better survival having lower unstaged disease. For patients with poor prognosis, cancer staging may not be necessary. On the other hand, not receiving a tumor stage may result in not receiving life extending treatment.

Strengths and weaknesses of the SEER data need some consideration. Cancer registries in the SEER Program incorporate several quality assurance measures, identify nearly all diagnosed cases in their catchment areas, and have a very high level of follow-up for vital status. Criteria used by SEER regarding formatting and defining case information are described elsewhere [25,26]. SEER data provide a large number of cases with detailed information on patient demographics, tumor characteristics, morphology, diagnostic information, and extent of disease. This allowed us to adjust for selected factors while assessing unstaged disease. In addition, the 17 SEER registries included in this study cover approximately 26% of the U.S., with both urban and rural areas represented. Hence, the results have a high level of external validity. One study found that the SEER coverage area may under represent tobacco-related cancers [27]. While SEER does not collect data on comorbid diseases for conditions other than cancer, relative survival provided us with a measure of net survival (survival in the absence of other causes).

## Conclusion

Poor health status, higher levels of comorbid diseases, greater difficulty in obtaining health insurance and more inoperable tumors associated with advancing age may have precluded some patients from obtaining tumor staging, which in turn limited treatment options and survival. These conditions may also have been more common in unmarried patients, thereby explaining their higher levels of unstaged disease. For 10 of the 18 cancer sites considered, blacks experienced higher levels of unstaged disease. Males had a lower level of unstaged disease for four of the cancer sites but a higher level of unstaged disease for two of the cancer sites. A significant decrease in unstaged disease occurred for 15 of the 18 cancer sites considered. Understanding reasons for a decrease in unstaged disease did not occur for all of the cancer sites considered. Further studies should explore reasons for differential levels of staging between whites and blacks, males and females for several of the cancer sites.

## Conflict of interest

The authors declare that they have no competing interests.

## Authors' contributions

RM conceived of the study, participated in the design of the study, and performed the statistical analysis. AS, AA, and KR carried out the literature review for the study and assisted in creating the tables. All authors assisted in writing the paper and have read and approved the final manuscript.

## Pre-publication history

The pre-publication history for this paper can be accessed here:

http://www.biomedcentral.com/1471-2407/11/402/prepub

## References

[B1] Yancik R Wesley MN Ries LA Effect of age and comorbidity in postmenopausal breast cancer patients aged 55 years and older JAMA 2001 285 7 885 892 10.1001/jama.285.7.885 11180731

[B2] Klassen AC Curriero F Kulldorf M Missing stage and grade in Maryland prostate cancer surveillance data, 1992-1997 Am J Prev Med 2006 30 2 Suppl S77 S87 16458794 10.1016/j.amepre.2005.09.010

[B3] Koroukian SM Xu F Beaird H Complexity of care needs and unstaged cancer in elders: a population-based study Cancer Detect Prev 2007 31 3 199 206 10.1016/j.cdp.2007.04.002 17658225 PMC2577170

[B4] Worthington JL Koroukian SM Cooper GS Examining the characteristics of unstaged colon and rectal cancer cases Cancer Detect Prev 2008 32 3 251 258 10.1016/j.cdp.2008.08.006 18804920

[B5] Bradley CJ Lin C Absence of cancer diagnosis and treatment in elderly Medicaid-insured nursing home residents J Natl Cancer Inst 2008 100 21 31 10.1093/jnci/djm271 18159068

[B6] Roetzheim RG Pal N Tennant C Effects of health insurance and race on early detection of cancer J Natl Cancer Inst 1999 91 16 1409 1415 10.1093/jnci/91.16.1409 10451447

[B7] Hankey BF Riew LA Edwards BK The Surveillance, Epidemiology and End Results program: a national resource Cancer Epidemiol Biomarkers Prev 1999 8 1117 1121 10613347

[B8] Ries LAG Reichman ME Lewis DR Hankey BF Edwards BK Cancer survival and incidence from the Surveillance, Epidemiology, and End Results (SEER) Program Oncologist 2003 8 541 552 10.1634/theoncologist.8-6-541 14657533

[B9] Percy C Van Holten V Muir C eds International Classification of Disease for Oncology, Second Edition 1990 Geneva: World Health Organization

[B10] Ries LAG Kosary CL Lyles BA Cancer patient survival: why use the relative survival rate The Abstract 1995 21 28 30

[B11] Henson DE Ries LA The relative survival rate Cancer 1995 76 1687 1688 10.1002/1097-0142(19951115)76:10<1687::AID-CNCR2820761002>3.0.CO;2-I 8625034

[B12] Percy CL Miller BA Ries LA Davis DL, Hoel D eds Effect of changes in cancer classification and the accuracy of cancer death certificates on trends in cancer mortality Trends in Cancer Mortality in Industrial Countries 1990 609 New York: New York Academy of Sciences 87 99 10.1111/j.1749-6632.1990.tb32059.x 2264660

[B13] Mettlin CJ Murphy GP Cunningham MP Menck HR The national cancer data base report on race, age, and region variations in prostate cancer treatment Cancer 1987 80 7 1261 1266 9317177

[B14] Patel MK Patel DA Lu M Elshaikh MA Munkarah A Movsas B Impact of marital status on survival among women with invasive cervical cancer: analysis of population-based Surveillance, Epidemiology, and End Results data J Low Genit Tract Dis 2010 14 4 329 338 10.1097/LGT.0b013e3181ddfa68 20885161

[B15] Van Jaarsveld CH Miles A Edwards R Wardle J Marriage and cancer prevention: does marital status and inviting both spouses together influence colorectal cancer screening participation? J Med Screen 2006 13 4 172 176 17217605 10.1177/096914130601300403

[B16] Gore JL Kwan L Saigal CS Litwin MS Marriage and mortality in bladder carcinoma Cancer 2005 104 6 1188 1194 10.1002/cncr.21295 16078264

[B17] Goodwin JS Hunt WC Key CR Samet JM The effect of marital status on stage, treatment, and survival of cancer patients JAMA 1987 258 3125 3130 10.1001/jama.258.21.3125 3669259

[B18] Sato N Ito Y Ioka A Tanaka M Tsukuma H Sex differences in stomach cancer survival in Osaka, Japan: analyses using relative survival model Jpn J Clin Oncol 2009 39 10 690 694 10.1093/jjco/hyp084 19687052

[B19] McCann Sex differences in cancer that don't make sense: or do they? J Natl Cancer Inst 2009 92 19 1560 1562 10.1093/jnci/92.19.1560 11018089

[B20] Miller BA Hankey BF Thomas TL Impact of sociodemographic factors, hormone receptor status, and tumor grade on ethnic differences in tumor stage and size for breast cancer in US Women Am J Epidemiol 2001 155 6 534 545 10.1093/aje/155.6.534 11882527

[B21] Putt M Long JA Montagnet C Racial differences in the impact of comorbidities on survival among elderly men with prostate cancer Med Care Res Rev 2009 66 4 409 435 10.1177/1077558709333996 19357389 PMC2780425

[B22] Chan JK Wu H Cheung MK Shin JY Osann K Kapp DS The outcomes of 27,063 women with unstage endometrioid uterine cancer Gynecol Oncol 2007 106 2 282 288 10.1016/j.ygyno.2007.05.033 17662377

[B23] Schwartz KL Crossley-May H Vigneau FD Brown K Banerjee M Race, socioeconomic status and stage at diagnosis for five common malignancies Cancer Causes Control 2003 14 8 761 766 10.1023/A:1026321923883 14674740

[B24] Calle EE Flanders WD Thun MJ Martin LM Demographic predictors of mammography and pap smear screening in US women Am J Public Health 1993 83 1 53 8417607 10.2105/ajph.83.1.53 PMC1694510

[B25] Ries L Fritz A The SEER Program Code Manual. Third Edition Surveillance Program Bethesda, MD: Division of Cancer Control and Population Sciences, National Cancer Institute, National Institutes of Health 1998

[B26] North American Association of Central Cancer Registries Standards for Cancer Registries. Volume III: Standards for Completeness, Quality, Analysis, and Management of Data 2000

[B27] Merrill RM Dearden KA How representative are the Surveillance, Epidemiology, and End Results (SEER) program cancer data of the United States? Cancer Causes Control 2004 15 1027 1034 10.1007/s10552-004-1324-5 15801487

